# Lack of Major Involvement of Common *CYP2C* Gene Polymorphisms in the Risk of Developing Cross-Hypersensitivity to NSAIDs

**DOI:** 10.3389/fphar.2021.648262

**Published:** 2021-09-21

**Authors:** Yolanda Macías, Jesús M. García-Menaya, Manuel Martí, Concepción Cordobés, Raquel Jurado-Escobar, José A. Cornejo-García, María J. Torres, Natalia Blanca-López, Gabriela Canto, Miguel Blanca, José J. Laguna, Joan Bartra, Ana Rosado, Javier Fernández, Elena García-Martín, José A. G. Agúndez

**Affiliations:** ^1^ University Institute of Molecular Pathology Biomarkers, UEx, Cáceres, Spain; ^2^ ARADyAL Instituto de Salud Carlos III, Cáceres, Spain; ^3^ Allergy Service, Badajoz University Hospital, Badajoz, Spain; ^4^ ARADyAL Instituto de Salud Carlos III, Badajoz, Spain; ^5^ Research Laboratory, IBIMA, Regional University Hospital of Málaga, UMA, Málaga, Spain; ^6^ ARADyAL Instituto de Salud Carlos III, Málaga, Spain; ^7^ Allergy Unit, IBIMA, Regional University Hospital of Málaga, UMA, Málaga, Spain; ^8^ Allergy Service, Infanta Leonor University Hospital, Madrid, Spain; ^9^ ARADyAL Instituto de Salud Carlos III, Madrid, Spain; ^10^ Allergy Unit and Allergy-Anaesthesia Unit, Hospital Central Cruz Roja, Faculty of Medicine, Alfonso X El Sabio University, Madrid, Spain; ^11^ Allergy Section, Pneumology Department, Hospital Clinic, ARADyAL, Universitat de Barcelona, Barcelona, Spain; ^12^ ARADyAL Instituto de Salud Carlos III, Barcelona, Spain; ^13^ Allergy Service, Alcorcón Hospital, Madrid, Spain; ^14^ Allergy Unit, Regional University Hospital, Alicante, Spain; ^15^ ARADyAL Instituto de Salud Carlos III, Alicante, Spain

**Keywords:** CYP2C8, CYP2C9, CYP2C19, NSAID, polymorphisms, hypersensitivity

## Abstract

Cross-hypersensitivity to non-steroidal anti-inflammatory drugs (NSAIDs) is a relatively common, non-allergic, adverse drug event triggered by two or more chemically unrelated NSAIDs. Current evidence point to COX-1 inhibition as one of the main factors in its etiopathogenesis. Evidence also suggests that the risk is dose-dependent. Therefore it could be speculated that individuals with impaired NSAID biodisposition might be at increased risk of developing cross-hypersensitivity to NSAIDs. We analyzed common functional gene variants for *CYP2C8, CYP2C9,* and *CYP2C19* in a large cohort composed of 499 patients with cross-hypersensitivity to NSAIDs and 624 healthy individuals who tolerated NSAIDs. Patients were analyzed as a whole group and subdivided in three groups according to the main enzymes involved in the metabolism of the culprit drugs as follows: CYP2C9, aceclofenac, indomethacin, naproxen, piroxicam, meloxicam, lornoxicam, and celecoxib; CYP2C8 plus CYP2C9, ibuprofen and diclofenac; CYP2C19 plus CYP2C9, metamizole. Genotype calls ranged from 94 to 99%. No statistically significant differences between patients and controls were identified in this study, either for allele frequencies, diplotypes, or inferred phenotypes. After patient stratification according to the enzymes involved in the metabolism of the culprit drugs, or according to the clinical presentation of the hypersensitivity reaction, we identified weak significant associations of a lower frequency (as compared to that of control subjects) of CYP2C8*3/*3 genotypes in patients receiving NSAIDs that are predominantly CYP2C9 substrates, and in patients with NSAIDs-exacerbated cutaneous disease. However, these associations lost significance after False Discovery Rate correction for multiple comparisons. Taking together these findings and the statistical power of this cohort, we conclude that there is no evidence of a major implication of the major functional *CYP2C* polymorphisms analyzed in this study and the risk of developing cross-hypersensitivity to NSAIDs. This argues against the hypothesis of a dose-dependent COX-1 inhibition as the main underlying mechanism for this adverse drug event and suggests that pre-emptive genotyping aiming at drug selection should have a low practical utility for cross-hypersensitivity to NSAIDs.

## Introduction

Nonsteroidal anti-inflammatory drugs (NSAIDs) are the most common drugs used to relieve pain and to decrease inflammation and fever. NSAIDs are among the most used drugs in the world, and they comprise a wide range of chemically unrelated compounds. It is estimated that over 30 million people worldwide use these medications daily, not only as prescription drugs but also over-the-counter (OTC) (Singh, 2000). As a general rule, prescription NSAIDs are effective to relieve chronic musculoskeletal pain and inflammation, and OTC NSAIDs, normally at lower doses, are effective to relieve acute or minor aches and pains. These drugs are quite safe, but despite being available OTC in many countries, they could bring about adverse drug reactions (ADRs).

ADRs have been reported in approximately 1.5% of the patients with at least one NSAIDs prescribed and around 23.3% of the adverse drug reactions to NSAIDs are due to hypersensitivity reactions (Blumenthal et al., 2016, 2017). One specific type of ADRs, hypersensitivity drug reactions (HDRs) can be divided into two main groups: on one side those which are initiated by specific immunological mechanisms (also described as drug allergy), the response is induced by a single drug, and patients are classified as selective drug responders. These reactions can be IgE-mediated, designated as immediate drug allergy, or T cell-mediated, designated as delayed drug allergy. On the other side, HDRs whose mechanisms are nonimmunological (also described as nonallergic hypersensitivity), the reaction is induced by two or more chemically unrelated drugs, and patients are classified as cross-intolerant or cross-hypersensitivity subjects (Johansson et al., 2004; Szczeklik et al., 2009; Doña et al., 2011).

According to their clinical presentation, cross-hypersensitivity reactions could be classified as NSAIDs-exacerbated respiratory disease (NERD), NSAIDs-exacerbated cutaneous disease (NECD), and NSAID-induced urticaria/angioedema (NIUA) (Kowalski et al., 2013). These non-immunological reactions are believed to be originated via inhibition of cyclooxygenase 1 (COX-1) enzyme and the release of histamine and sulphidopeptide leukotrienes (Kowalski et al., 2007; Doña et al., 2018; Bakhriansyah et al., 2019; Li and Laidlaw, 2019; Mastalerz et al., 2019). In this context, it is important to bear in mind that NSAIDs antagonize inflammation by interfering with the function of cyclooxygenases, and therefore their association with nonallergic hypersensitivity might be related to disequilibrium in the arachidonic acid degradation pathways, that is, interference with the formation of prostaglandins and thromboxanes, thus resulting in the shunting of arachidonic acid metabolism towards the 5-lipoxygenase pathway, and the consequent increase in the release of cysteinyl leukotrienes (Sánchez-Borges, 2010; Caimmi et al., 2012).

Interindividual variability in drug metabolism is likely to be involved in HDRs (Agúndez et al., 2015a, Agúndez et al., 2018; García-Martín et al., 2015; Ariza et al., 2016; Sánchez-Gómez et al., 2016; Plaza-Serón et al., 2018). A substantial part of such interindividual variability is associated with polymorphisms in genes coding drug-metabolizing enzymes. NSAIDs are extensively metabolized by Cytochrome P450 2C enzymes (CYP2C) and *CYP2C* gene variants are strongly related to the pharmacokinetics, pharmacological effects, and adverse drug reactions for many NSAIDs (Agúndez JA. et al., 2009; Agúndez et al., 2009 J.; Agúndez et al., 2011; Szczeklik et al., 2009; Martínez et al., 2014; Macías et al., 2020; Theken et al., 2020). Impaired CYP2C metabolism brings about decreased clearance, increased drug exposure, and therefore, increased COX-inhibition. Since cross-hypersensitivity induced by NSAIDs is believed to be related to COX-inhibition, it is conceivable that individuals with genetic alterations leading to impairment in NSAID metabolism would be more prone to developing cross-hypersensitivity induced by these drugs. However, no studies have been conducted to test such a hypothesis. We analyzed such putative association in a large study group with enough sample size to support or discard a major association between common *CYP2C* functional gene variants and the risk of developing cross-hypersensitivity with NSAIDs metabolized by these enzymes.

## Methods

### Participants

A total cohort of 1.123 participants was analyzed in this study, all were Spanish individuals with South European Ancestry. Ancestry was self-reported. Four hundred and ninety-nine patients who developed hypersensitivity to acetylsalicylic acid (ASA) and one or more chemically different NSAIDs mainly metabolized by CYP2C enzymes were included in the study. Their mean age was 42 (SD = 17.46) years. Also, six hundred and twenty-four healthy individuals with an average age of 21 (SD = 2.32) years participated as control subjects. All control individuals tolerated NSAIDs that are CYP2C substrates. Patients and controls were recruited between 2007 and 2020 from the Allergy Services of the following hospitals in Spain: Badajoz University Hospital, Málaga University Hospital, Madrid Cruz Roja Hospital, Barcelona Clinic Hospital, Madrid Infanta Leonor Hospital, Alcorcón University Hospital, and Elche University Hospital. Control individuals were selected among the staff and students assessed through anamnesis, clinical history and/or self-reported tolerance to COX-1 inhibitors. Inclusion criteria for the patients were as follows: Diagnosis of cross-hypersensitivity (Pérez-Alzate et al., 2017; Blanca-López et al., 2018, Blanca-López et al., 2019) by clinical history and a positive drug provocation test, for one or more of the following NSAIDs: ibuprofen, diclofenac, aceclofenac, indomethacin, naproxen, piroxicam, meloxicam, lornoxicam, celecoxib, and metamizole. ASA-positivity was included as a requisite in the diagnosis because in cross-reactive (non-allergic) hypersensitivity patients react to all strong COX-1 inhibitors, including ASA, whereas allergic hypersensitivity patients tolerate ASA (Kowalski et al., 2013; Pérez-Alzate et al., 2017; Angeletti et al., 2020); besides, CYP2C9 plays a role in ASA metabolism (Thiessen, 1983; Hutt et al., 1986; Bigler et al., 2001; Palikhe et al., 2011; Gómez-Tabales et al., 2020). Patients who presented with hypersensitivity triggered by other NSAIDs whose metabolism is not mainly catalyzed by CYP2C enzymes (including clonixinate, dexketoprofen, ketorolac, etofenamate, ketoprofen, piketoprofen, propifenazone, phenylbutazone, aminophenazone, acetaminophen, etoricoxib and oxyphenbutazone) were excluded from the study. The study was conducted according to the principles of the Declaration of Helsinki and approved by the Ethics Committees of each participating hospital. Written informed consent was obtained from all the participants involved in the study.

**FIGURE 1:**
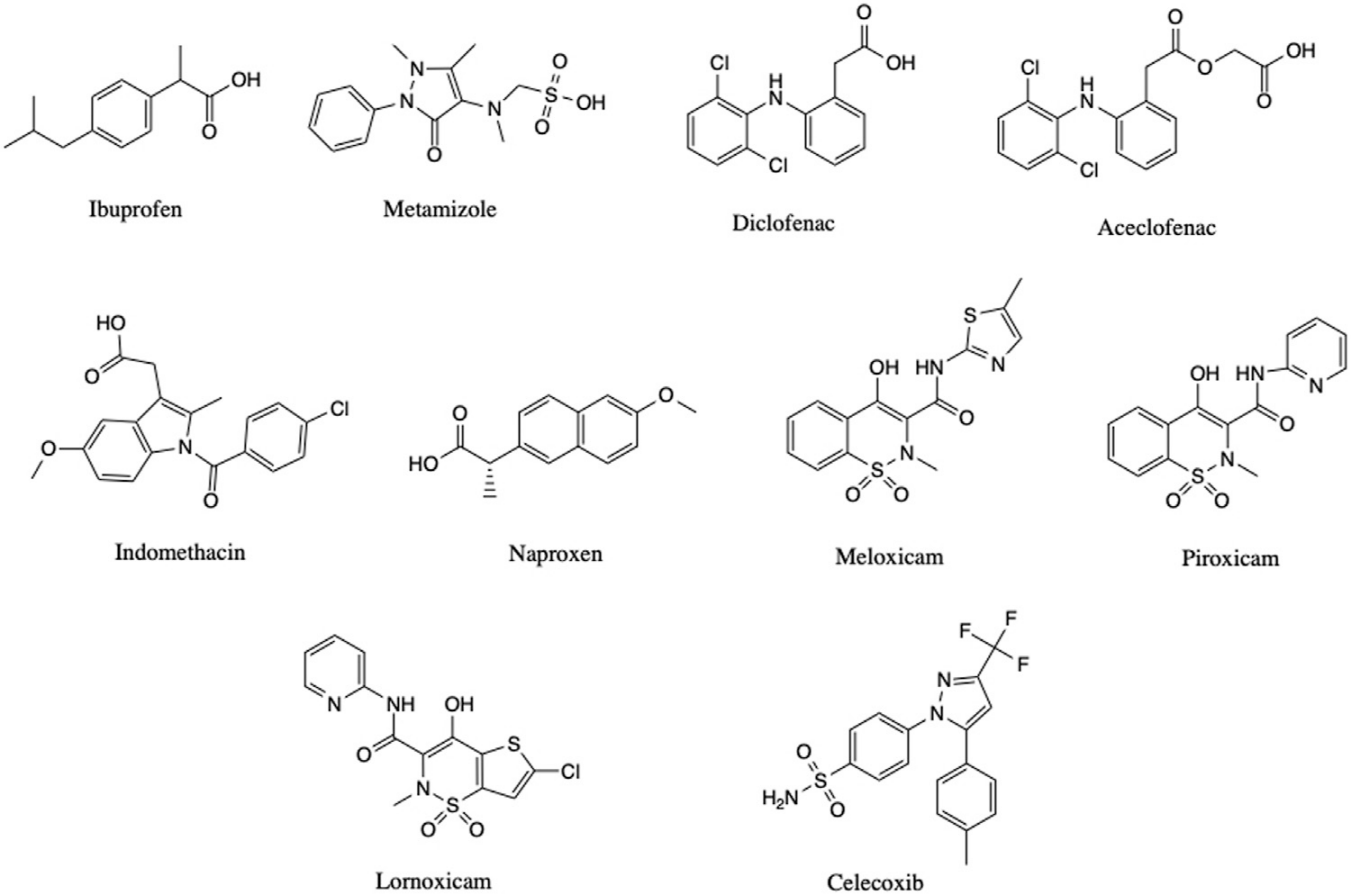
Drug structures.

The main NSAIDs (Figure 1) that triggered the hypersensitivity reaction are shown in Table 1. The clinical presentations stratified according to the culprit drugs involved are summarized in Table 2.

**TABLE 1:** Characteristics of the individuals and drug involved in NSAID-induced cross-hypersensitivity in this study.

	Total N	Men N (%)	Women N (%)
Controls	624 (55.57)	225 (51.84)	399 (57.91)
Patients	499 (44.43)	209 (48.16)	290 (42.09)
**Culprit drug**	**Total N (%)**	**Men N (%)**	**Women N (%)**
Ibuprofen	353 (45.43)	145 (45.03)	208 (45.71)
Metamizole	246 (31.66)	104 (32.30)	142 (31.21)
Diclofenac	108 (13.90)	45 (13.98)	63 (13.85)
Naproxen	36 (4.63)	15 (4.66)	21 (4.62)
Aceclofenac	12 (1.54)	5 (1.55)	7 (1.54)
Piroxicam	11 (1.42)	3 (0.93)	8 (1.76)
Indomethacin	5 (0.64)	3 (0.93)	2 (0.44)
Meloxicam	3 (0.39)	1 (0.31)	2 (0.44)
Lornoxicam	2 (0.26)	0	2 (0.44)
Celecoxib	1 (0.13)	1 (0.31)	0
Total^a^	777 (100)	322 (100)	455 (100)

a The total number exceeds the number of patients because many of them presented cross hypersensitivity to two or more drugs.

**TABLE 2:** Gender and clinical presentation of NSAID-induced cross-hypersensitivity in this study.

Gender	NECD N (%)	NERD N (%)	Mixed pattern N (%)	Anaphylaxis N (%)	NIUA N (%)	Unknown N (%)	Total N (%)
Men	144 (42.48)	22 (37.93)	22 (36.67)	17 (50.00)	1 (50.00)	3 (50.00)	209
Women	195 (57.52)	36 (62.07)	38 (63.33)	17 (50.00)	1 (50.00)	3 (50.00)	290
**Culprit drug**	**NECD N (%)**	**NERD N (%)**	**Mixed pattern N (%)**	**Anaphylaxis N (%)**	**NIUA N (%)**	**Unknown N (%)**	**Total N (%)**
Ibuprofen	254 (46.95)	36 (42.35)	42 (48.28)	19 (34.55)	2 (66.67)	2 (33.33)	355
Metamizole	165 (30.50)	33 (38.82)	23 (26.44)	22 (40.00)	0 (0.00)	1 (16.67)	244
Diclofenac	73 (13.49)	8 (9.41)	15 (17.24)	9 (16.36)	1 (33.33)	2 (33.33)	108
Aceclofenac	11 (2.03)	1 (1.18)	0 (0.00)	0 (0.00)	0 (0.00)	0 (0.00)	12
Indomethacin	2 (0.37)	2 (2.35)	1 (1.15)	0 (0.00)	0 (0.00)	0 (0.00)	5
Naproxen	24 (4.44)	4 (4.71)	3 (3.45)	5 (9.09)	0 (0.00)	0 (0.00)	36
Meloxicam	2 (0.37)	1 (1.18)	0 (0.00)	0 (0.00)	0 (0.00)	0 (0.00)	3
Piroxicam	9 (1.66)	0 (0.00)	1 (1.15)	0 (0.00)	0 (0.00)	1 (16.67)	11
Lornoxicam	0 (0.00)	0 (0.00)	2 (2.30)	0 (0.00)	0 (0.00)	0 (0.00)	2
Celecoxib	1 (0.18)	0 (0.00)	0 (0.00)	0 (0.00)	0 (0.00)	0 (0.00)	1
Total^a^	541 (100)	85 (100)	87 (100)	55 (100)	3 (100)	6 (100)	

a The total number exceeds the number of patients because many of them presented cross hypersensitivity to two or more drugs.

### Genotyping Study

Genomic DNA was obtained and purified by following standard procedures and then genotypic analyses were performed using a real-time quantitative polymerase chain reaction (qPCR). The target SNVs were selected according to their functional effect or clinical implications, as well as the signature allele frequencies in the population studied. The analyses focused on the signature SNVs for Tier 1 variant alleles according to the PharmVar database (https://www.pharmvar.org/). For *CYP2C9*, Tier 1 alleles are CYP2C9*2, *3, *5, *6, *8, and *11 (Pratt et al., 2019). Among these, the alleles CYP2C9*5, *6, *8, and *11 were not included in the analyses because their signature SNVs had extremely low frequencies (ranging from 0.00002 to 0.003) in European individuals according to public databases (https://gnomad.broadinstitute.org). Therefore, we analyzed CYP2C9*2 (rs1799853) and CYP2C9*3 (rs1057910). Regarding *CYP2C19*, Tier 1 alleles are the *CYP2C19* alleles *2 (rs12769205), *3 (rs4986893) and *17 (rs12248560) (Botton et al., 2020; Pratt et al., 2018). CYP2C19*3 allele was excluded from the study because its signature SNV has a very low allele frequency (equal to 0.0003) in European individuals (Botton et al., 2020). Although no Tier 1 variants have been defined for *CYP2C*8, we used the same criteria as reported elsewhere (Pratt et al., 2018, Pratt et al., 2019), based on their reported clinical relevance, CYP2C8-associated medications, and their frequency. We selected the variant alleles CYP2C8*3 (rs11572080) and *4 (rs1058930). CYP2C8*2 (rs11572103) was not included because its signature SNV has a very low frequency among Europeans (0.003). All SNVs were tested by using TaqMan Assays (Life Sciences, Alcobendas, Madrid, Spain) pre-designed to detect the above-mentioned SNVs. Details of the TaqMan probes and minor allele frequencies in the study population are shown in Table 3. Assignment of predicted phenotype based on genotype was carried out as described elsewhere for *CYP2C9* (Theken et al., 2020), and *CYP2C19* (Lima et al., 2020). For *CYP2C8*, predicted phenotype was carried out assuming that both, CYP2C8*3 and CYP2C8*4 alleles, lead to decreased metabolic activity (Bahadur et al., 2002).

**TABLE 3:** SNVs analyzed in this study.

Allele	dbSNP	Chromosomal location	Minor allele frequency (control subjects)	Statistical power (one tailed/two tailed; OR = 1.5, *α* = 0.0083) (%)
CYP2C8*3	rs11572080 C/T	10:96827030	0.1615	89.02/83.74
CYP2C8*4	rs1058930 G/C	10:96818119	0.0611	50.65/41.03^a^ ^(1)^
CYP2C9*2	rs1799853 C/T	10:96702047	0.1558	88.09/82.53
CYP2C9*3	rs1057910 A/C	10:96741053	0.0701	56.73/47.07^a^ ^(2)^
CYP2C19*2	rs12769205 A/G	10:96535124	0.1424	85.52/79.26
CYP2C19*17	rs12248560 C/T	10:96521657	0.2104	94.27/90.88

a *The statistical power* (*one tailed/two tailed, OR* = *1.8, α* = *0.0083*) *is: (1) 88.85%/83.54%; (2) 92.59%/88.54%*.

### Statistical Analyses

The R package SNPassoc (González et al., 2014) was used to calculate allele and genotypic frequencies, to determine the Hardy-Weinberg’s equilibrium (HWE) using the exact test as described elsewhere (Wigginton et al., 2005) and to analyze differences between groups (González et al., 2007).

The comparison of the frequencies of each SNV between traits was performed by using binary logistic regression, assuming different genetic models. To evaluate the genotype risks we applied a traditional logistic regression establishing the most frequent level (*1/*1) as the baseline. The model also includes sex as a predictor and offers two different adjusted *p*-values from the likelihood ratio test (LRT). The specific *p*-value measures the significance of the risk of a particular level with respect to the *1/*1 diplotype while the global *p*-value measures whether the inclusion of all diplotypes different to *1/*1 as a predictor brings about significant additional information about the trait.

When assessing allele risks, the additive model (each copy of the minor allele modifies the risk in an additive form) was used. The analysis was carried out generating a numeric feature with value 0 for the baseline (wild type), 1 for heterozygous for defect alleles, and 2 for homozygous. The additive model was also applied to measure the risk of inferred phenotypes. For *CYP2C8* and *CYP2C9* the baseline level is RM, while for *CYP2C19* we have established NM as baseline level, IM as intermediate high risk level, PM as higher risk level, RM as intermediate low risk level and UR as lower risk level.

The *p*-values (Tables 4, 5) were adjusted by gender and were obtained by likelihood ratio test (LRT), comparing the likelihood of the nested model that only includes gender as predictor, with the least restrictive model that includes gender and alleles or inferred genotype as predictor.

**TABLE 4:** Alleles, genotypes and inferred phenotypes observed in overall patients and healthy controls.

Alleles	Patients (No)	Patients (%)	Controls (No)	Controls (%)	OR (adjusted): Wald	Intergroup comparison values. *p*-value (adjusted): LRT global
CYP2C8*3 C/C	350	71.87%	434	71.15%	0.9 (0.71–1.14)	0.372
CYP2C8*3 C/T	130	26.69%	155	25.41%		
CYP2C8*3 T/T	7	1.44%	21	3.44%		
Total	487		610			
CYP2C8*4 G/G	433	89.83%	520	88.29%	0.85 (0.59–1.22)	0.378
CYP2C8*4 C/G	47	9.75%	66	11.21%		
CYP2C8*4 C/C	2	0.41%	3	0.51%		
Total	482		589			
CYP2C9*2 C/C	365	73.89%	441	71.94%	0.88 (0.7–1.12)	0.293
CYP2C9*2 C/T	120	24.29%	153	24.96%		
CYP2C9*2 T/T	9	1.82%	19	3.10%		
Total	494		613			
CYP2C9*3 A/A	410	87.23%	513	86.66%	0.96 (0.69–1.34)	0.812
CYP2C9*3 A/C	57	12.13%	75	12.67%		
CYP2C9*3 C/C	3	0.64%	4	0.68%		
Total	470		592			
CYP2C19*2 A/A	367	73.84%	449	72.65%	1.00 (0.78–1.28)	0.995
CYP2C19*2 A/G	119	23.94%	162	26.21%		
CYP2C19*2 G/G	11	2.21%	7	1.13%		
Total	497		618			
CYP2C19*17 C/C	312	65.00%	376	62.77%	0.93 (0.75–1.14)	0.483
CYP2C19*17 C/T	146	30.42%	194	32.39%		
CYP2C19*17 T/T	22	4.58%	29	4.84%		
**TOTAL**	**480**		**599**			

**Genotypes**	**Patients (No)**	**Patients (%)**	**Controls (No)**	**Controls (%)**	**OR (adjusted)**	**Intergroup comparison values. *p*-value (adjusted): LRT global**
CYP2C8*1/*1	304	64.27%	351	60.52%	—	0.152
CYP2C8*1/*3	115	24.31%	140	24.14%	0.95 (0.71–1.27)	
CYP2C8*1/*4	34	7.19%	55	9.48%	0.71 (0.45–1.12)	
CYP2C8*3/*3	7	1.48%	21	3.62%	0.37 (0.15–0.88)	
CYP2C8*3/*4	11	2.33%	10	1.72%	1.27 (0.53–3.05)	
CYP2C8*4/*4	2	0.42%	3	0.52%	0.72 (0.12–4.35)	
Total	**473**		**580**			
CYP2C9*1/*1	301	64.45%	354	60.10%	—	0.550
CYP2C9*1/*2	100	21.41%	137	23.26%	0.85 (0.63–1.15)	
CYP2C9*1/*3	47	10.06%	62	10.53%	0.92 (0.6–1.36)	
CYP2C9*2/*2	8	1.71%	19	3.23%	0.5 (0.21–1.15)	
CYP2C9*2/*3	8	1.71%	13	2.21%	0.73 (0.3–1.79)	
CYP2C9*3/*3	3	0.64%	4	0.68%	0.81 (0.18–3.68)	
Total	**467**		**589**			
CYP2C19*1/*1	211	44.05%	248	41.61%	—	0.463
CYP2C19*1/*2	92	19.21%	120	20.13%	0.89 (0.64–1.24)	
CYP2C19*1/*17	118	24.63%	155	26.01%	0.9 (0.66–1.21)	
CYP2C19*2/*2	11	2.30%	6	1.01%	2.3 (0.83–6.34)	
CYP2C19*2/*17	26	5.43%	40	6.71%	0.77 (0.45–1.31)	
CYP2C19*17/*17	21	4.38%	27	4.53%	0.89 (0.49–1.63)	
Total	**479**		**596**			
**Inferred Phenotypes**	**Patients (No)**	**Patients (%)**	**Controls (No)**	**Controls (%)**	**OR (adjusted)**	**Intergroup comparison values. *p*-value (adjusted): LRT global**
CYP2C8 RM	304	64.27%	351	60.52%	0.85 (0.69–1.05)	0.126
CYP2C8 IM	149	31.50%	195	33.62%		
CYP2C8 PM	20	4.23%	34	5.86%		
Total	**473**		**580**			
*CYP2C9* RM	301	64.45%	354	60.10%	0.85 (0.67–1.06)	0.144
CYP2C9 IM	155	33.19%	218	37.01%		
CYP2C9 PM	11	2.36%	17	2.89%		
Total	**467**		**589**			
CYP2C19 UR	21	4.38%	27	4.53%	1.03 (0.9–1.19)	0.664
CYP2C19 RM	118	24.63%	155	26.01%		
CYP2C19 NM	211	44.05%	248	41.61%		
CYP2C19 IM	118	24.63%	160	26.85%		
CYP2C19 PM	11	2.30%	6	1.01%		
Total	**479**		**596**			

IM, intermediate metabolizer; LTR, likelihood ratio test; NM, normal metabolizer; No, number; OR, odds ratio; PM, poor metabolizer; RM, rapid metabolizer; UR = ultrarapid metabolizer.

**TABLE 5:** Alleles, genotypes and inferred phenotypes observed in the three subgroups of patients.

Alleles	Patients group 1 (No)	Patients (%)	OR (adjusted)	Intergroup comparison values. *p*-value (adjusted): LRT global	Patients group 2 (No)	Patients (%)	OR (adjusted)	Intergroup comparison values. *p*-value (adjusted): LRT global	Patients group 3 (No)	Patients (%)	OR (adjusted)	Intergroup comparison values. *p*-value (adjusted): LRT global
CYP2C8*3 C/C	48	71.64%	0.86 (0.52–1.42)	0.546	277	72.32%	0.89 (0.69–1.14)	0.353	171	71.55%	0.9 (0.67–1.21)	0.488
CYP2C8*3 C/T	19	28.36%			100	26.11%			65	27.20%		
CYP2C8*3 T/T	0	0.00%			6	1.57%			3	1.26%		
**Total**	**67**				**383**				**239**			
CYP2C8*4 G/G	61	89.71%	0.82 (0.37–1.81)	0.616	341	89.50%	0.89 (0.6–1.31)	0.550	215	90.72%	0.76 (0.47–1.23)	0.259
CYP2C8*4 C/G	7	10.29%			38	9.97%			21	8.86%		
CYP2C8*4 C/C	0	0.00%			2	0.52%			1	0.42%		
**Total**	**68**				**381**				**237**			
CYP2C9*2 C/C	47	70.15%	0.95 (0.58–1.56)	0.845	288	73.85%	0.88 (0.69–1.14)	0.332	179	73.97%	0.90 (0.68–1.21)	0.496
CYP2C9*2 C/T	20	29.85%			95	24.36%			57	23.55%		
CYP2C9*2 T/T	0	0.00%			7	1.79%			6	2.48%		
**Total**	**67**				**390**				**242**			
CYP2C9*3 A/A	54	83.08%	1.24 (0.65–2.38)	0.526	322	87.03%	0.97 (0.68–1.39)	0.875	199	86.90%	0.98 (0.64–1.5)	0.925
CYP2C9*3 A/C	11	16.92%			46	12.43%			29	12.66%		
CYP2C9*3 C/C	0	0.00%			2	0.54%			1	0.44%		
**Total**	**65**				**370**				**229**			
CYP2C19*2 A/A	47	70.15%	1.2 (0.73–1.99)	0.479	293	74.74%	0.96 (0.74–1.25)	0.783	181	73.88%	1.01 (0.74–1.37)	0.952
CYP2C19*2 A/G	18	26.87%			90	22.96%			58	23.67%		
CYP2C19*2 G/G	2	2.99%			9	2.30%			6	2.45%		
**Total**	**67**				**392**				**245**			
CYP2C19*17 C/C	40	59.70%	1.12 (0.73–1.7)	0.610	254	67.20%	0.87 (0.69–1.09)	0.216	152	64.41%	0.93 (0.71–1.21)	0.585
CYP2C19*17 C/T	23	34.33%			107	28.31%			74	31.36%		
CYP2C19*17 T/T	4	5.97%			17	4.50%			10	4.24%		
	**67**				**378**				**236**			
												

Genotypes	Patients Group 1 (No)	Patients (%)	OR (adjusted)	Intergroup comparison values. p-value (adjusted): LRT global	Patients Group 2 (No)	Patients (%)	OR (adjusted)	Intergroup comparison values. p-value (adjusted): LRT global	Patients Group 3 (No)	Patients (%)	OR (adjusted)	Intergroup comparison values. p-value (adjusted): LRT global
CYP2C8*1/*1	45	67.16%	—	0.043	240	64.34%	—	0.277	148	64.63%	—	0.372
CYP2C8*1/*3	15	22.39%	0.84 (0.45–1.56)		89	23.86%	0.93 (0.68–1.27)		56	24.45%	1.00 (0.69–1.43)	
CYP2C8*1/*4	3	4.48%	0.42 (0.13–1.39)		27	7.24%	0.71 (0.44–1.17)		18	7.86%	0.75 (0.42–1.32)	
CYP2C8*3/*3			0 (0-inf)		6	1.61%	0.4 (0.16–1.01)		3	1.31%	0.32 (0.09–1.1)	
CYP2C8*3/*4	4	5.97%	3.01 (0.9–10.03)		9	2.41%	1.32 (0.53–3.3)		3	1.31%	0.69 (0.19–2.54)	
CYP2C8*4/*4			0 (0-inf)		2	0.54%	0.92 (0.15–5.59)		1	0.44%	0.75 (0.08–7.35)	
**Total**	**67**				**373**				**232**			
CYP2C9*1/*1	35	54.69%	—	0.261	236	64.13%	—	0.528	147	65.33%	—	0.843
CYP2C9*1/*2	18	28.13%	1.33 (0.73–2.43)		80	21.74%	0.87 (0.63–1.2)		43	19.11%	0.82 (0.56–1.21)	
CYP2C9*1/*3	9	14.06%	1.5 (0.68–3.27)		39	10.60%	0.96 (0.62–1.48)		24	10.67%	0.91 (0.55–1.53)	
CYP2C9*2/*2	0	0.00%	0 (0-inf)		6	1.63%	0.47 (0.19–1.2)		4	1.78%	0.63 (0.23–1.73)	
CYP2C9*2/*3	2	3.13%	1.59 (0.34–7.32)		5	1.36%	0.59 (0.21–1.69)		7	3.11%	1.13 (0.42–3.04)	
CYP2C9*3/*3	0	0.00%	0 (0-inf)		2	0.54%	0.71 (0.13–3.92)		0	0.00%	0.57 (0.06–5.15)	
**Total**	**64**				**368**				**229**			
CYP2C19*1/*1	24	36.36%	—	0.808	178	47.21%	—	0.217	99	41.95%	—	0.378
CYP2C19*1/*2	14	21.21%	1.19 (0.59–2.39)		69	18.30%	0.8 (0.56–1.13)		49	20.76%	1.02 (0.68–1.53)	
CYP2C19*1/*17	19	28.79%	1.26 (0.67–2.38)		85	22.55%	0.77 (0.55–1.07)		63	26.69%	1.01 (0.69–1.47)	
CYP2C19*2/*2	2	3.03%	3.71 (0.7–19.57)		9	2.39%	2.22 (0.77–6.36)		6	2.54%	2.61 (0.82–8.32)	
CYP2C19*2/*17	4	6.06%	1.03 (0.34–3.14)		20	5.31%	0.7 (0.4–1.24)		9	3.81%	0.58 (0.27–1.22)	
CYP2C19*17/*17	3	4.55%	1.08 (0.3–3.85)		16	4.24%	0.81 (0.42–1.56)		10	4.24%	0.9 (0.42–1.93)	
**Total**	**66**				**377**				**236**			
												

Inferred Phenotypes	Patients Group 1 (No)	Patients (%)	OR (adjusted)	Intergroup comparison values. p-value (adjusted): LRT global	Patients Group 2 (No)	Patients (%)	OR (adjusted)	Intergroup comparison values. p-value (adjusted): LRT global	Patients Group 3 (No)	Patients (%)	OR (adjusted)	Intergroup comparison values. p-value (adjusted): LRT global
CYP2C8 RM	45	67.16%	0.82 (0.53–1.27)	0.372	241	64.44%	0.86 (0.69–1.07)	0.177	148	64.63%	0.82 (0.63–1.07)	0.144
CYP2C8 IM	18	26.87%			116	31.02%			74	32.31%		
CYP2C8 PM	4	5.97%			17	4.55%			7	3.06%		
**Total**	**67**				**373**				**232**			
CYP2C9 RM	35	54.69%	1.2 (0.76–1.9)	0.429	236	64.84%	0.84 (0.66–1.08)	0.167	147	65.33%	0.88 (0.66–1.17)	0.372
CYP2C9 IM	27	42.19%			123	33.79%			72	32.00%		
CYP2C9 PM	2	3.13%			5	1.37%			6	2.67%		
**Total**	**64**				**368**				**229**			
CYP2C19 UR	3	4.55%	1.03 (0.77–1.38}	0.843	16	4.24%	1.05 (0.9–1.22)	0.517	10	4.24%	1.02 (0.85–1.21)	0.857
CYP2C19 RM	19	28.79%			85	22.55%			63	26.69%		
CYP2C19 NM	24	36.36%			178	47.21%			99	41.95%		
CYP2C19 IM	18	27.27%			89	23.61%			58	24.58%		
CYP2C19 PM	2	3.03%			9	2.39%			6	2.54%		
**Total**	**66**				**377**				**236**			

IM, intermediate metabolizer; LTR, likelihood ratio test; NM, normal metabolizer; No, number; OR, odds ratio; PM, poor metabolizer; RM, rapid metabolizer; UR = ultrarapid metabolizer.

The results were considered statistically significant when *p-*value*s* were equal or under 0.05. Also, the odds ratio (OR) of Wald Test associated was estimated with a 95% confidence interval (CI). False Discovery Rate (FDR) correction was used for multiple comparison adjustments (Benjamini et al., 2001).

The statistical power for the sample size of this study was calculated to analyze the minor allele frequency (MAF) with a genetic model with an odds ratio value = 1.5 determined from the allele frequencies observed in healthy subjects in previous studies carried out in Spaniards (García-Martín et al., 2001, García-Martín et al., 2004, García-Martín et al., 2015; Alonso-Navarro et al., 2006; Blanco et al., 2008; Ladero et al., 2012; Martínez et al., 2014). The Bonferroni correction was used to make an adjustment for multiple comparisons: the significance level of 0.05 was reduced (*α* = 0.0083) according to the number of comparisons made (6 in this study). The statistical power for all cases and each SNV analyzed is detailed in Table 3. For most SNVs the statistical power was high enough to detect an OR = 1.5 with a bilateral power higher than 80%. For two SNVs (rs1058930 and rs1057910), because of the low MAF observed in this study, the statistical power was not sufficient to detect an OR = 1.5, but it was sufficient to detect an OR = 1.8 (Table 3).

## Results

The most common drugs involved for cross-reactive hypersensitivity induced by NSAIDs were ibuprofen, metamizole and diclofenac (Table 1), and the most frequent clinical presentation was NECD (Table 2). Since many patients experienced cross-reactive hypersensitivity with more than one drug, the sum of the patients in each subgroup in these Tables exceeds the total number of patients. The clinical presentation was strongly related to the drug involved. NECD was particularly related to ibuprofen, whereas anaphylaxis was mainly related to metamizole (Table 2).

To determine the influence of genetic polymorphisms in the risk of developing cross-reactive hypersensitivity, genotypes, diplotypes, and inferred phenotypes were compared in overall patients and healthy controls (Table 4). Genotype calls were very high for all SNVs as follows: CYP2C8*3: 97.6% patients and 97.8% controls; CYP2C8*4: 96.6% patients and 94.4% controls; CYP2C9*2: 99.0% patients and 98.2% controls; CYP2C9*3: 94.2% patients and 94.9% controls; CYP2C19*2: 99.6% patients and 99.0% controls; and CYP2C19*17: 96.2% patients and 96.0% controls. All SNVs were at Hardy-Weinberg’s equilibrium in patients and control individuals and the allele frequencies correspond with those previously described in Spaniards (Martínez et al., 2001; García-Martín et al., 2006; Blanco et al., 2008; Martínez et al., 2014; García-Martín et al., 2015), as well as those reported in public databases (https://gnomad.broadinstitute.org). For individuals who were successfully genotyped for all relevant SNVs in each gene, inferred phenotypes were calculated from diplotypes as described under Methods. The percentage of individuals with inferred phenotypes were as follows: *CYP2C8*: 95.0% patients and 92.9% controls; *CYP2C9*: 93.2% patients and 94.4% controls; and *CYP2C19*: 95.8% patients and 95.5% controls. No statistically significant differences either in allele frequencies, genotypes, or inferred phenotypes were observed when comparing patients with control individuals (Table 4), thus suggesting that CYP2C-related impaired NSAID metabolism is not strongly related to the risk of developing cross-hypersensitivity to NSAIDs.

Since the role of CYP2C enzymes in the metabolism of NSAIDs vary depending on the substrate, patients were divided into three subgroups according to the main enzymes involved in the metabolism of the culprit drugs: group 1: drugs mainly metabolized by CYP2C9 (aceclofenac, indomethacin, naproxen, piroxicam, meloxicam, lornoxicam, and celecoxib); group 2: drugs mainly metabolized by CYP2C8 and CYP2C9 (ibuprofen and diclofenac); and group 3: drugs mainly metabolized by CYP2C19 and CYP2C9 (metamizole) (Leemann et al., 1993; Bonnabry et al., 1996; Miners et al., 1996; Türck et al., 1996; Hamman et al., 1997; Chesné et al., 1998; FDA (Food and Drug Administration), 1998; Skjodt and Davies, 1998; Bort et al., 1999; Davies and Skjodt, 1999; Davies et al., 2000; Henrotin et al., 2001; Tang et al., 2001; Martínez et al., 2005; Perini et al., 2005; Tornio et al., 2007; Chang et al., 2008; Agúndez JA. et al., 2009; Byrav et al., 2009; Neunzig et al., 2012; Abdalla et al., 2014; Martínez et al., 2014; Lucas, 2016; ). Table 5 shows the genotyping and inferred phenotype results. Again, no statistically significant differences were observed between any of the patient’s subgroups and control individuals.

The only statistically significant difference observed was in the subgroup of patients with cross-hypersensitivity to drugs that are predominantly CYP2C9 substrates although the significance was weak and it was related to the *CYP2C8* genotypes (Table 5). This difference (*p* = 0.043) is attributable to a lower frequency of carriers of CYP2C8*3/*3 among patients as compared to control individuals. However, such a difference was not statistically significant after FDR correction (*p* = 0.129). When patients were stratified according to the clinical presentation (Supplementary Tables S1–S4), the only statistically significant difference was related to a low frequency of NECD patients homozygous for the CYP2C8*3 allele, as compared to healthy individuals (*p* = 0.029). However, the statistical significance disappeared after FDR correction (*p* = 0.174).

## Discussion

The role of COX-1 inhibition in the etiopathogenesis of cross-hypersensitivity to NSAIDs has been the object of controversy for years (Kowalski et al., 2007; Doña et al., 2018; Mastalerz et al., 2019). Supporting this hypothesis, it has been shown that COX-2 inhibitors are well tolerated among patients with cross-hypersensitivity to NSAIDs (Morales et al., 2014; Bakhriansyah et al., 2019) and that patients with *PTGS1* gene variants related to a decreased activity (Agúndez et al., 2014; Agúndez et al., 2015b; Lucena et al., 2019) are at increased risk of developing cross-hypersensitivity to NSAIDs (García-Martín et al., 2021). Interestingly, preliminary evidence suggests that cross-hypersensitivity to NSAIDs is dose-dependent (Palmer, 2005; Kong et al., 2007; Kowalski et al., 2013; Blumenthal et al., 2017) and, therefore, it could be speculated that individuals with impaired NSAID clearance (and therefore increased drug exposure) might have increased risk of developing cross-hypersensitivity. This hypothesis, however, was not investigated in detail. Preliminary studies have shown the lack of association of Aspirin Induced Asthma and *CYP2C19* genotypes (Kooti et al., 2020), which is not surprising since CYP2C19 is not relevant in aspirin metabolism. This aside, no studies have been conducted to assess the putative role of impaired NSAID metabolism in the risk of developing cross-hypersensitivity to NSAIDs.

Strengths in this study include a large sample of patients with cross-reactive hypersensitivity induced to NSAID (n = 499). This sample size allows a good statistical power. A limitation of this study is that plasma levels of the NSAIDs and metabolites could not be obtained because the serum of patients during the acute phase was not available. Therefore, the putative association between genotypes and plasma levels could not be ascertained. Nevertheless, it is widely accepted that the genetic variants analyzed in this study are strongly related to pharmacokinetic changes, and several clinical practice guidelines on CYP2C enzymes (all based on the potential of gene variants to induce pharmacokinetic changes in drugs known to be CYP2C substrates) have been published (Johnson et al., 2011, Johnson et al., 2017; Caudle et al., 2014; Hicks et al., 2017; Moriyama et al., 2017; Karnes et al., 2020; Lima et al., 2020; Theken et al., 2020; Westergaard et al., 2020). Another limitation is that treatment regimen was not specifically recorded, although usually the hypersensitivity reaction occurs after a single standard dose of the corresponding NSAID.

The results of this study do not support a major association between common *CYP2C* gene variants leading to altered NSAID metabolism and the risk of developing cross-hypersensitivity to NSAIDs. These findings are unexpected if the hypothesis of a putative dose-dependent COX-1 inhibition as a major factor in the development of cross-hypersensitivity is correct. However, the high sample size and the statistical power obtained in this study rule out a major association. It cannot be ruled out putative associations with very rare detrimental allelic variants that have not been analyzed here because of the extremely low frequencies, however, the lack of association with common detrimental alleles observed in this study makes it very unlikely that such putative associations with rare alleles might exist.

It is to be noted that all cases involved ASA, and that therefore, our conclusions are valid only for patients with cross-hypersensitivity involving ASA. CYP2C enzymes play a minor role in ASA metabolism (Agúndez et al., 2009). However, CYP2C9 plays a major role in the metabolism of salicylic acid to gentisic acid (Gómez-Tabales et al., 2020). Also, CYP2C9 is involved in the production of NADPH-dependent hydrogen peroxide in the presence of salicylic acid. Therefore, although the role of CYP2C9 in ASA biodisposition might be quantitatively small, a role in adverse reactions due to ASA cannot be ruled out.

The findings obtained in this study argue against the hypothesis of a dose-dependent (in this case a drug exposure-dependent) COX-1 inhibition as a relevant mechanism in cross-hypersensitivity to NSAIDs and, therefore, will add to the controversy of the mechanisms underlying the development of cross-hypersensitivity to NSAIDs. The main clinical implication of our findings is that we found no evidence supporting the utility of pre-emptive *CYP2C* genotyping aiming at drug selection for patients with a previous history of cross-hypersensitivity to NSAIDs. However, the findings obtained in this study do not rule out the potential of pharmacogenetics testing combined with phenotyping factors and testing for other genes involved in NSAID pharmacodynamics and/or genes involved in the development and the clinical presentation of the hypersensitivity reactions, such as genes related to the arachidonic acid pathway, as well as those related to inflammation mediators, and oxidative stress.

## Data Availability

The datasets presented in this study can be found in online repositories. The names of the repository/repositories and accession number(s) can be found in the article/Supplementary Material.
