# Supplementary material for: Lack of Major Involvement of Common CYP2C Gene Polymorphisms in the Risk of Developing Cross-Hypersensitivity to NSAIDs
Source: Front Pharmacol. 2021 Sep 21;12:648262. doi: 10.3389/fphar.2021.648262 (PMC8490926; doi:10.3389/fphar.2021.648262)
Supplement: Supplementary file 1 [file DataSheet1.docx]

**Supplemental Table S1. Alleles, genotypes and inferred phenotypes observed in patients with NECD and control individuals.**

| **Alleles** | **NECD (No)** | **NECD (%)** | **Controls (No)** | **Controls (%)** | **OR**  **(adjusted):**  **Wald** | **Intergroup comparison values.**  **p-value (adjusted):**  **LRT global** |
| --- | --- | --- | --- | --- | --- | --- |
| CYP2C8*3 C/C | 242 | 72.89% | 434 | 71.15% | − | 0.029 |
| CYP2C8*3 C/T | 87 | 26.20% | 155 | 25.41% | 1.01 (0.74-1.37) |  |
| CYP2C8*3 T/T | 3 | 0.90% | 21 | 3.44% | 0.24 (0.07-0.83) |  |
| **Total** | **332** |  | **610** |  |  |  |
| CYP2C8*4 G/G | 303 | 90.72% | 520 | 88.29% | − | 0.452 |
| CYP2C8*4 C/G | 29 | 8.68% | 66 | 11.21% | 0.75 (0.47-1.18) |  |
| CYP2C8*4 C/C | 2 | 0.60**%** | 3 | 0.51% | 1.07 (0.18-6.47) |  |
| **Total** | **334** |  | **589** |  |  |  |
| CYP2C9*2 C/C | 248 | 73.81**%** | 441 | 71.94% | − | 0.064 |
| CYP2C9*2 C/T | 85 | 25.30**%** | 153 | 24.96% | 0.99 (0.72-1.34) |  |
| CYP2C9*2 T/T | 3 | 0.89**%** | 19 | 3.10% | 0.28 (0.08-0.95) |  |
| **Total** | **336** |  | **613** |  |  |  |
| CYP2C9*3 A/A | 280 | 87.50**%** | 513 | 86.66% | − | 0.954 |
| CYP2C9*3 A/C | 38 | 11.88**%** | 75 | 12.67% | 0.95 (0.62-1.44) |  |
| CYP2C9*3 C/C | 2 | 0.63**%** | 4 | 0.68% | 0.85 (0.15-4.71) |  |
| **Total** | **320** |  | **592** |  |  |  |
| CYP2C19*2 A/A | 245 | 72.49**%** | 449 | 72.65% | − | 0.673 |
| CYP2C19*2 A/G | 87 | 25.74**%** | 162 | 26.21% | 0.98 (0.73-1.33) |  |
| CYP2C19*2 G/G | 6 | 1.78**%** | 7 | 1.13% | 1.65 (0.55-4.97) |  |
| **Total** | **338** |  | **618** |  |  |  |
| CYP2C19*17 C/C | 217 | 65.76**%** | 376 | 62.77% | − | 0.648 |
| CYP2C19*17 C/T | 99 | 30.00**%** | 194 | 32.39% | 0.89 (0.66-1.19) |  |
| CYP2C19*17 T/T | 14 | 4.24**%** | 29 | 4.84% | 0.82 (0.42-1.59) |  |
| **Total** | **330** |  | **599** |  |  |  |
|  |  |  |  |  |  |  |
| **Genotypes** | **NECD (No)** | **NECD (%)** | **Controls (No)** | **Controls (%)** | **OR**  **(adjusted):**  **Wald** | **Intergroup comparison values.**  **p-value (adjusted):**  **LRT global** |
| CYP2C8*1/*1 | 215 | 65.55% | 351 | 60.52% | − | 0.065 |
| CYP2C8*1/*3 | 80 | 24.39% | 140 | 24.14% | 0.94 (0.68-1.3) |  |
| CYP2C8*1/*4 | 22 | 6.71% | 55 | 9.48% | 0.65 (0.38-1.1) |  |
| CYP2C8*3/*3 | 3 | 0.91% | 21 | 3.62% | 0.22 (0.06-0.75) |  |
| CYP2C8*3/*4 | 6 | 1.83% | 10 | 1.72% | 0.98 (0.35-2.75) |  |
| CYP2C8*4/*4 | 2 | 0.61% | 3 | 0.52% | 1.01 (0.17-6.14) |  |
| **Total** | **328** |  | **580** |  |  |  |
| CYP2C9*1/*1 | 202 | 63.72% | 354 | 60.10% | − | 0.247 |
| CYP2C9*1/*2 | 73 | 23.03% | 137 | 23.26% | 0.93 (0.67-1.3) |  |
| CYP2C9*1/*3 | 33 | 10.41% | 62 | 10.53% | 0.95 (0.6-1.5) |  |
| CYP2C9*2/*2 | 3 | 0.95% | 19 | 3.23% | 0.27 (0.08-0.94) |  |
| CYP2C9*2/*3 | 4 | 1.26% | 13 | 2.21% | 0.55 (0.18-1.71) |  |
| CYP2C9*3/*3 | 2 | 0.63% | 4 | 0.68% | 0.82 (0.15-4.53) |  |
| **Total** | **317** |  | **589** |  |  |  |
| CYP2C19*1/*1 | 146 | 44.51% | 248 | 41.61% | − | 0.759 |
| CYP2C19*1/*2 | 66 | 20.12% | 120 | 20.13% | 0.93 (0.64-1.33) |  |
| CYP2C19*1/*17 | 77 | 23.48% | 155 | 26.01% | 0.84 (0.6-1.19) |  |
| CYP2C19*2/*2 | 6 | 1.83% | 6 | 1.01% | 1.82 (0.57-5.77) |  |
| CYP2C19*2/*17 | 20 | 6.10% | 40 | 6.71% | 0.85 (0.48-1.51) |  |
| CYP2C19*17/*17 | 13 | 3.96% | 27 | 4.53% | 0.79 (0.39-1.59) |  |
| **Total** | **328** |  | **596** |  |  |  |
|  |  |  |  |  |  |  |
| **Inferred Phenotypes** | **NECD (No)** | **NECD (%)** | **Controls (No)** | **Controls (%)** | **OR**  **(adjusted):**  **Wald** | **Intergroup comparison values.**  **p-value (adjusted):**  **LRT global** |
| CYP2C8 RM | 215 | 65.55% | 351 | 60.52% | − | 0.107 |
| CYP2C8 IM | 102 | 31.10% | 195 | 33.62% | 0.86 (0.64-1.15) |  |
| CYP2C8 PM | 11 | 3.35% | 34 | 5.86% | 0.51 (0.25-1.03) |  |
| **Total** | **328** |  | **580** |  |  |  |
| CYP2C9 RM | 202 | 63.72% | 354 | 60.10% | − | 0.436 |
| CYP2C9 IM | 109 | 34.38% | 218 | 37.01% | 0.88 (0.66-1.17) |  |
| CYP2C9 PM | 6 | 1.89% | 17 | 2.89% | 0.62 (0.24-1.59) |  |
| **Total** | **317** |  | **589** |  |  |  |
| CYP2C19 UR | 13 | 3.96% | 27 | 4.53% | 0.79 (0.39-1.59) | 0.638 |
| CYP2C19 RM | 77 | 23.48% | 155 | 26.01% | 0.84 (0.6-1.19) |  |
| CYP2C19 NM | 146 | 44.51% | 248 | 41.61% | − |  |
| CYP2C19 IM | 86 | 26.22% | 160 | 26.85% | 0.91 (0.65-1.27) |  |
| CYP2C19 PM | 6 | 1.83% | 6 | 1.01% | 1.82 (0.57-5.77) |  |
| **Total** | **328** |  | **596** |  |  |  |

**Supplemental Table S2. Alleles, genotypes and inferred phenotypes observed in patients with NERD and control individuals.**

| **Alleles** | **NERD (No)** | **NERD (%)** | **Controls (No)** | **Controls (%)** | **OR**  **(adjusted):**  **Wald** | **Intergroup comparison values.**  **p-value (adjusted):**  **LRT global** |
| --- | --- | --- | --- | --- | --- | --- |
| CYP2C8*3 C/C | 39 | 68.42% | 434 | 71.15% | − | 0.601 |
| CYP2C8*3 C/T | 17 | 29.82% | 155 | 25.41% | 1.23 (0.67-2.23) |  |
| CYP2C8*3 T/T | 1 | 1.75% | 21 | 3.44% | 0.52 (0.07-3.99) |  |
| **Total** | **57** |  | **610** |  |  |  |
| CYP2C8*4 G/G | 48 | 87.27% | 520 | 88.29% | − | 0.732 |
| CYP2C8*4 C/G | 7 | 12.73% | 66 | 11.21% | 1.14 (0.49-2.62) |  |
| CYP2C8*4 C/C | 0 | 0.00% | 3 | 0.51% | 0 (0-Inf) |  |
| **Total** | **55** |  | **589** |  |  |  |
| CYP2C9*2 C/C | 41 | 70.69% | 441 | 71.94% | − | 0.975 |
| CYP2C9*2 C/T | 15 | 25.86% | 153 | 24.96% | 1.06 (0.57-1.96) |  |
| CYP2C9*2 T/T | 2 | 3.45% | 19 | 3.10% | 1.14 (0.26-5.05) |  |
| **Total** | **58** |  | **613** |  |  |  |
| CYP2C9*3 A/A | 44 | 80.00% | 513 | 86.66% | − | 0.365 |
| CYP2C9*3 A/C | 10 | 18.18% | 75 | 12.67% | 1.59 (0.76-3.3) |  |
| CYP2C9*3 C/C | 1 | 1.82% | 4 | 0.68% | 2.79 (0.3-25.61) |  |
| **Total** | **55** |  | **592** |  |  |  |
| CYP2C19*2 A/A | 49 | 84.48% | 449 | 72.65% | − | 0.084 |
| CYP2C19*2 A/G | 8 | 13.79% | 162 | 26.21% | 0.45 (0.21-0.98) |  |
| CYP2C19*2 G/G | 1 | 1.72% | 7 | 1.13% | 1.31 (0.16-10.85) |  |
| **Total** | **58** |  | **618** |  |  |  |
| CYP2C19*17 C/C | 31 | 55.36% | 376 | 62.77% | − | 0.550 |
| CYP2C19*17 C/T | 22 | 39.29% | 194 | 32.39% | 1.38 (0.78-2.44) |  |
| CYP2C19*17 T/T | 3 | 5.36% | 29 | 4.84% | 1.24 (0.36-4.32) |  |
| **Total** | **56** |  | **599** |  |  |  |
|  |  |  |  |  |  |  |
| **Genotypes** | **NERD (No)** | **NERD (%)** | **Controls (No)** | **Controls (%)** | **OR**  **(adjusted):**  **Wald** | **Intergroup comparison values.**  **p-value (adjusted):**  **LRT global** |
| CYP2C8*1/*1 | 33 | 61.11% | 351 | 60.52% | − | 0.463 |
| CYP2C8*1/*3 | 14 | 25.93% | 140 | 24.14% | 1.07 (0.56-2.06) |  |
| CYP2C8*1/*4 | 3 | 5.56% | 55 | 9.48% | 0.58 (0.17-1.94) |  |
| CYP2C8*3/*3 | 1 | 1.85% | 21 | 3.62% | 0.5 (0.06-3.81) |  |
| CYP2C8*3/*4 | 3 | 5.56% | 10 | 1.72% | 3.15 (0.82-12.02) |  |
| CYP2C8*4/*4 | 0 | 0.00% | 3 | 0.52% | 0 (0-Inf) |  |
| **Total** | **54** |  | **580** |  |  |  |
| CYP2C9*1/*1 | 32 | 58.18% | 354 | 60.10% | − | 0.737 |
| CYP2C9*1/*2 | 12 | 21.82% | 137 | 23.26% | 0.97 (0.49-1.94) |  |
| CYP2C9*1/*3 | 6 | 10.91% | 62 | 10.53% | 1.09 (0.44-2.72) |  |
| CYP2C9*2/*2 | 1 | 1.82% | 19 | 3.23% | 0.59 (0.08-4.52) |  |
| CYP2C9*2/*3 | 3 | 5.45% | 13 | 2.21% | 2.62 (0.71-9.7) |  |
| CYP2C9*3/*3 | 1 | 1.82% | 4 | 0.68% | 2.66 (0.29-24.59) |  |
| **Total** | **55** |  | **589** |  |  |  |
| CYP2C19*1/*1 | 23 | 41.07% | 248 | 41.61% | − | 0.219 |
| CYP2C19*1/*2 | 7 | 12.50% | 120 | 20.13% | 0.63 (0.26-1.5) |  |
| CYP2C19*1/*17 | 21 | 37.50% | 155 | 26.01% | 1.46 (0.78-2.73) |  |
| CYP2C19*2/*2 | 1 | 1.79% | 6 | 1.01% | 1.81 (0.21-15.67) |  |
| CYP2C19*2/*17 | 1 | 1.79% | 40 | 6.71% | 0.27 (0.04-2.05) |  |
| CYP2C19*17/*17 | 3 | 5.36% | 27 | 4.53% | 1.18 (0.33-4.2) |  |
| **Total** | **56** |  | **596** |  |  |  |
|  |  |  |  |  |  |  |
| **Inferred Phenotypes** | **NERD (No)** | **NERD (%)** | **Controls (No)** | **Controls (%)** | **OR**  **(adjusted):**  **Wald** | **Intergroup comparison values.**  **p-value (adjusted):**  **LRT global** |
| CYP2C8 RM | 33 | 61.11% | 351 | 60.52% | − | 0.892 |
| CYP2C8 IM | 17 | 31.48% | 195 | 33.62% | 0.93 (0.5-1.71) |  |
| CYP2C8 PM | 4 | 7.41% | 34 | 5.86% | 1.23 (0.41-3.7) |  |
| **Total** | **54** |  | **580** |  |  |  |
| CYP2C9 RM | 32 | 58.18% | 354 | 60.10% | − | 0.301 |
| CYP2C9 IM | 19 | 34.55% | 218 | 37.01% | 0.97 (0.54-1.76) |  |
| CYP2C9 PM | 4 | 7.27% | 17 | 2.89% | 2.63 (0.83-8.29) |  |
| **Total** | **55** |  | **589** |  |  |  |
| CYP2C19 UR | 3 | 5.36% | 27 | 4.53% | 1.18 (0.33-4.2) | 0.179 |
| CYP2C19 RM | 21 | 37.50% | 155 | 26.01% | 1.46 (0.78-2.73) |  |
| CYP2C19 NM | 23 | 41.07% | 248 | 41.61% | − |  |
| CYP2C19 IM | 8 | 14.29% | 160 | 26.85% | 0.54 (0.23-1.23) |  |
| CYP2C19 PM | 1 | 1.79% | 6 | 1.01% | 1.81 (0.21-15.67) |  |
| **Total** | **56** |  | **596** |  |  |  |

**Supplemental Table S3. Alleles, genotypes and inferred phenotypes observed in patients with mixed pattern and control individuals.**

| **Alleles** | **Mixed pattern (No)** | **Mixed pattern (%)** | **Controls (No)** | **Controls (%)** | **OR**  **(adjusted):**  **Wald** | **Intergroup comparison values.**  **p-value (adjusted):**  **LRT global** |
| --- | --- | --- | --- | --- | --- | --- |
| CYP2C8*3 C/C | 43 | 74.14% | 434 | 71.15% | − | 0.879 |
| CYP2C8*3 C/T | 13 | 22.41% | 155 | 25.41% | 0.85 (0.44-1.62) |  |
| CYP2C8*3 T/T | 2 | 3.45% | 21 | 3.44% | 0.95 (0.21-4.2) |  |
| **Total** | **58** |  | **610** |  |  |  |
| CYP2C8*4 G/G | 50 | 89.29% | 520 | 88.29% | − | 0.754 |
| CYP2C8*4 C/G | 6 | 10.71% | 66 | 11.21% | 0.94 (0.39-2.27) |  |
| CYP2C8*4 C/C | 0 | 0.00% | 3 | 0.51% | 0 (0-Inf) |  |
| **Total** | **56** |  | **589** |  |  |  |
| CYP2C9*2 C/C | 46 | 79.31% | 441 | 71.94% | − | 0.399 |
| CYP2C9*2 C/T | 10 | 17.24% | 153 | 24.96% | 0.63 (0.31-1.27) |  |
| CYP2C9*2 T/T | 2 | 3.45% | 19 | 3.10% | 1.01 (0.23-4.47) |  |
| **Total** | **58** |  | **613** |  |  |  |
| CYP2C9*3 A/A | 50 | 89.29% | 513 | 86.66% | − | 0.635 |
| CYP2C9*3 A/C | 6 | 10.71% | 75 | 12.67% | 0.84 (0.35-2.02) |  |
| CYP2C9*3 C/C | 0 | 0.00% | 4 | 0.68% | 0 (0-Inf) |  |
| **Total** | **56** |  | **592** |  |  |  |
| CYP2C19*2 A/A | 43 | 72.88% | 449 | 72.65% | − | 0.127 |
| CYP2C19*2 A/G | 13 | 22.03% | 162 | 26.21% | 0.84 (0.44-1.6) |  |
| CYP2C19*2 G/G | 3 | 5.08% | 7 | 1.13% | 4.54 (1.13-18.27) |  |
| **Total** | **59** |  | **618** |  |  |  |
| CYP2C19*17 C/C | 39 | 67.24% | 376 | 62.77% | − | 0.753 |
| CYP2C19*17 C/T | 16 | 27.59% | 194 | 32.39% | 0.8 (0.43-1.46) |  |
| CYP2C19*17 T/T | 3 | 5.17% | 29 | 4.84% | 0.98 (0.29-3.38) |  |
| **Total** | **58** |  | **599** |  |  |  |
|  |  |  |  |  |  |  |
| **Genotypes** | **Mixed pattern (No)** | **Mixed pattern (%)** | **Controls (No)** | **Controls (%)** | **OR**  **(adjusted):**  **Wald** | **Intergroup comparison values.**  **p-value (adjusted):**  **LRT global** |
| CYP2C8*1/*1 | 36 | 65.45% | 351 | 60.52% | − | 0.952 |
| CYP2C8*1/*3 | 11 | 20.00% | 140 | 24.14% | 0.77 (0.38-1.55) |  |
| CYP2C8*1/*4 | 5 | 9.09% | 55 | 9.48% | 0.88 (0.33-2.34) |  |
| CYP2C8*3/*3 | 2 | 3.64% | 21 | 3.62% | 0.91 (0.2-4.06) |  |
| CYP2C8*3/*4 | 1 | 1.82% | 10 | 1.72% | 0.96 (0.12-7.76) |  |
| CYP2C8*4/*4 | 0 | 0.00% | 3 | 0.52% | 0 (0-Inf) |  |
| **Total** | **55** |  | **580** |  |  |  |
| CYP2C9*1/*1 | 39 | 70.91% | 354 | 60.10% | − | 0.316 |
| CYP2C9*1/*2 | 8 | 14.55% | 137 | 23.26% | 0.53 (0.24-1.16) |  |
| CYP2C9*1/*3 | 6 | 10.91% | 62 | 10.53% | 0.88 (0.36-2.17) |  |
| CYP2C9*2/*2 | 2 | 3.64% | 19 | 3.23% | 0.96 (0.21-4.26) |  |
| CYP2C9*2/*3 | 0 | 0.00% | 13 | 2.21% | 0 (0-Inf) |  |
| CYP2C9*3/*3 | 0 | 0.00% | 4 | 0.68% | 0 (0-Inf) |  |
| **Total** | **55** |  | **589** |  |  |  |
| CYP2C19*1/*1 | 26 | 44.83% | 248 | 41.61% | − | 0.391 |
| CYP2C19*1/*2 | 10 | 17.24% | 120 | 20.13% | 0.79 (0.37-1.7) |  |
| CYP2C19*1/*17 | 13 | 22.41% | 155 | 26.01% | 0.8 (0.4-1.6) |  |
| CYP2C19*2/*2 | 3 | 5.17% | 6 | 1.01% | 4.95 (1.16-21.14) |  |
| CYP2C19*2/*17 | 3 | 5.17% | 40 | 6.71% | 0.72 (0.21-2.48) |  |
| CYP2C19*17/*17 | 3 | 5.17% | 27 | 4.53% | 1.03 (0.29-3.65) |  |
| **Total** | **58** |  | **596** |  |  |  |
|  |  |  |  |  |  |  |
| **Inferred Phenotypes** | **Mixed pattern (No)** | **Mixed pattern (%)** | **Controls (No)** | **Controls (%)** | **OR**  **(adjusted):**  **Wald** | **Intergroup comparison values.**  **p-value (adjusted):**  **LRT global** |
| CYP2C8 RM | 36 | 65.45% | 351 | 60.52% | − | 0.762 |
| CYP2C8 IM | 16 | 29.09% | 195 | 33.62% | 0.8 (0.43-1.48) |  |
| CYP2C8 PM | 3 | 5.45% | 34 | 5.86% | 0.85 (0.25-2.9) |  |
| **Total** | **55** |  | **580** |  |  |  |
| CYP2C9 RM | 39 | 70.91% | 354 | 60.10% | − | 0.087 |
| CYP2C9 IM | 16 | 29.09% | 218 | 37.01% | 0.67 (0.36-1.22) |  |
| CYP2C9 PM | 0 | 0.00% | 17 | 2.89% | 0 (0-Inf) |  |
| **Total** | **55** |  | **589** |  |  |  |
| CYP2C19 UR | 3 | 5.17% | 27 | 4.53% | 1.03 (0.29-3.65) | 0.268 |
| CYP2C19 RM | 13 | 22.41% | 155 | 26.01% | 0.8 (0.4-1.6) |  |
| CYP2C19 NM | 26 | 44.83% | 248 | 41.61% | − |  |
| CYP2C19 IM | 13 | 22.41% | 160 | 26.85% | 0.77 (0.39-1.55) |  |
| CYP2C19 PM | 3 | 5.17% | 6 | 1.01% | 4.95 (1.16-21.15) |  |
| **Total** | **58** |  | **596** |  |  |  |

**Supplemental Table S4. Alleles, genotypes and inferred phenotypes observed in patients with anaphylaxis and control individuals.**

| **Alleles** | **Anaphylaxis (No)** | **Anaphylaxis (%)** | **Controls (No)** | **Controls (%)** | **OR**  **(adjusted):**  **Wald** | **Intergroup comparison values.**  **p-value (adjusted):**  **LRT global** |
| --- | --- | --- | --- | --- | --- | --- |
| CYP2C8*3 C/C | 21 | 65.63% | 434 | 71.15% | − | 0.187 |
| CYP2C8*3 C/T | 11 | 34.38% | 155 | 25.41% | 1.48 (0.7-3.15) |  |
| CYP2C8*3 T/T | 0 | 0.00% | 21 | 3.44% | 0 (0-Inf) |  |
| **Total** | **32** |  | **610** |  |  |  |
| CYP2C8*4 G/G | 24 | 82.76% | 520 | 88.29% | − | 0.625 |
| CYP2C8*4 C/G | 5 | 17.24% | 66 | 11.21% | 1.54 (0.57-4.2) |  |
| CYP2C8*4 C/C | 0 | 0.00**%** | 3 | 0.51% | 0 (0-Inf) |  |
| **Total** | **29** |  | **589** |  |  |  |
| CYP2C9*2 C/C | 25 | 73.53**%** | 441 | 71.94% | − | 0.644 |
| CYP2C9*2 C/T | 7 | 20.59**%** | 153 | 24.96% | 0.81 (0.34-1.91) |  |
| CYP2C9*2 T/T | 2 | 5.88**%** | 19 | 3.10% | 1.85 (0.41-8.44) |  |
| **Total** | **34** |  | **613** |  |  |  |
| CYP2C9*3 A/A | 29 | 90.63**%** | 513 | 86.66% | − | 0.732 |
| CYP2C9*3 A/C | 3 | 9.38**%** | 75 | 12.67% | 0.79 (0.23-2.67) |  |
| CYP2C9*3 C/C | 0 | 0.00**%** | 4 | 0.68% | 0 (0-Inf) |  |
| **Total** | **32** |  | **592** |  |  |  |
| CYP2C19*2 A/A | 25 | 73.53**%** | 449 | 72.65% | − | 0.666 |
| CYP2C19*2 A/G | 8 | 23.53**%** | 162 | 26.21% | 0.89 (0.39-2.01) |  |
| CYP2C19*2 G/G | 1 | 2.94**%** | 7 | 1.13% | 2.79 (0.33-23.81) |  |
| **Total** | **34** |  | **618** |  |  |  |
| CYP2C19*17 C/C | 20 | 71.43**%** | 376 | 62.77% | − | 0.457 |
| CYP2C19*17 C/T | 6 | 21.43**%** | 194 | 32.39% | 0.59 (0.23-1.49) |  |
| CYP2C19*17 T/T | 2 | 7.14**%** | 29 | 4.84% | 1.22 (0.27-5.5) |  |
| **Total** | **28** |  | **599** |  |  |  |
|  |  |  |  |  |  |  |
| **Genotypes** | **Anaphylaxis (No)** | **Anaphylaxis (%)** | **Controls (No)** | **Controls (%)** | **OR**  **(adjusted):**  **Wald** | **Intergroup comparison values.**  **p-value (adjusted):**  **LRT global** |
| CYP2C8*1/*1 | 15 | 53.57% | 351 | 60.52% | − | 0.597 |
| CYP2C8*1/*3 | 8 | 28.57% | 140 | 24.14% | 1.36 (0.56-3.28) |  |
| CYP2C8*1/*4 | 4 | 14.29% | 55 | 9.48% | 1.63 (0.52-5.12) |  |
| CYP2C8*3/*3 | 0 | 0.00% | 21 | 3.62% | 0 (0-Inf) |  |
| CYP2C8*3/*4 | 1 | 3.57% | 10 | 1.72% | 2.09 (0.25-17.62) |  |
| CYP2C8*4/*4 | 0 | 0.00% | 3 | 0.52% | 0 (0-Inf) |  |
| **Total** | **28** |  | **580** |  |  |  |
| CYP2C9*1/*1 | 23 | 69.70% | 354 | 60.10% | − | 0.687 |
| CYP2C9*1/*2 | 5 | 15.15% | 137 | 23.26% | 0.57 (0.21-1.52) |  |
| CYP2C9*1/*3 | 2 | 6.06% | 62 | 10.53% | 0.55 (0.13-2.41) |  |
| CYP2C9*2/*2 | 2 | 6.06% | 19 | 3.23% | 1.63 (0.36-7.49) |  |
| CYP2C9*2/*3 | 1 | 3.03% | 13 | 2.21% | 1.27 (0.16-10.21) |  |
| CYP2C9*3/*3 | 0 | 0.00% | 4 | 0.68% | 0 (0-Inf) |  |
| **Total** | **33** |  | **589** |  |  |  |
| CYP2C19*1/*1 | 13 | 44.83% | 248 | 41.61% | − | 0.686 |
| CYP2C19*1/*2 | 7 | 24.14% | 120 | 20.13% | 1.11 (0.43-2.85) |  |
| CYP2C19*1/*17 | 5 | 17.24% | 155 | 26.01% | 0.62 (0.22-1.78) |  |
| CYP2C19*2/*2 | 1 | 3.45% | 6 | 1.01% | 3.8 (0.42-34.69) |  |
| CYP2C19*2/*17 | 1 | 3.45% | 40 | 6.71% | 0.47 (0.06-3.73) |  |
| CYP2C19*17/*17 | 2 | 6.90% | 27 | 4.53% | 1.3 (0.28-6.12) |  |
| **Total** | **29** |  | **596** |  |  |  |
|  |  |  |  |  |  |  |
| **Inferred Phenotypes** | **Anaphylaxis (No)** | **Anaphylaxis (%)** | **Controls (No)** | **Controls (%)** | **OR**  **(adjusted):**  **Wald** | **Intergroup comparison values.**  **p-value (adjusted):**  **LRT global** |
| CYP2C8 RM | 15 | 53.57% | 351 | 60.52% | − | 0.537 |
| CYP2C8 IM | 12 | 42.86% | 195 | 33.62% | 1.44 (0.66-3.14) |  |
| CYP2C8 PM | 1 | 3.57% | 34 | 5.86% | 0.62 (0.08-4.89) |  |
| **Total** | **28** |  | **580** |  |  |  |
| CYP2C9 RM | 23 | 69.70% | 354 | 60.10% | − | 0.564 |
| CYP2C9 IM | 9 | 27.27% | 218 | 37.01% | 0.66 (0.3-1.45) |  |
| CYP2C9 PM | 1 | 3.03% | 17 | 2.89% | 0.94 (0.12-7.4) |  |
| **Total** | **33** |  | **589** |  |  |  |
| CYP2C19 UR | 2 | 6.90% | 27 | 4.53% | 1.3 (0.28-6.12) | 0.671 |
| CYP2C19 RM | 5 | 17.24% | 155 | 26.01% | 0.62 (0.22-1.78) |  |
| CYP2C19 NM | 13 | 44.83% | 248 | 41.61% | − |  |
| CYP2C19 IM | 8 | 27.59% | 160 | 26.85% | 0.95 (0.38-2.34) |  |
| CYP2C19 PM | 1 | 3.45% | 6 | 1.01% | 3.8 (0.42-34.69) |  |
| **Total** | **29** |  | **596** |  |  |  |
